# Development of 3D CAD/FEM Analysis System for Natural Teeth and Jaw Bone Constructed from X-Ray CT Images

**DOI:** 10.1155/2010/659802

**Published:** 2010-07-18

**Authors:** Aki Hasegawa, Akikazu Shinya, Yuji Nakasone, Lippo V. J. Lassila, Pekka K. Vallittu, Akiyoshi Shinya

**Affiliations:** ^1^Department of Crown and Bridge, School of Life Dentistry at Tokyo, The Nippon Dental University, 1-9-20 Fujimi, Chiyoda-Ku, Tokyo 102-8159, Japan; ^2^Department of Prosthetic Dentistry and Biomaterials Science, Institute of Dentistry, University of Turku, Lemminkäisenkatu 2, 20520 Turku, Finland; ^3^Department of Machanical Engineering, Tokyo University of Science, 1-14-6 Kudan-Kita, Chiyoda-Ku, Tokyo 102-0073, Japan

## Abstract

A three-dimensional finite element model of the lower first premolar, with the three layers of enamel, dentin, and pulp, and the mandible, with the two layers of cortical and cancellous bones, was directly constructed from noninvasively acquired CT images. This model was used to develop a system to analyze the stresses on the teeth and supporting bone structure during occlusion based on the finite element method and to examine the possibility of mechanical simulation.

## 1. Introduction

In medical and dental research, the strain gauge technique [1, 2], photoelastic test [3, 4], and finite element method [5–11] are commonly used to analyze the stress of structural objects with complex morphology. The strain gauge technique only allows for measurement of discontinuous surface areas and cannot provide measurements of internal stress. The photoelastic test can determine internal stress but construction of the model is difficult and its accuracy is limited. Since 1956, when the finite element method (FEM) was theoretically established by Turner et al. [12], FEM has been practically and broadly applied to the field of structural-mechanical analysis. FEM is a stress analysis technique used to determine overall stress and displacement by dividing the continuous region of the structural object into a finite number of elements and by calculating the dynamic equilibrium among these elements. In this regard, the FEM analysis has interested medical and dental researchers and is now one of the most successful engineering computational methods.

 In the dental field, a tooth and a bone are mainly important subjects and have very complicated structure. Generally, for three-dimensional (3D) FE modeling, several methods including those based on anatomical morphological means [13], measurement of dry skulls [14], and the coordinate transformation of data obtained by micro-CT and three-dimensional (3D) coordinate measuring machine [15, 16] are used. However, it is difficult to establish an accurate and valid 3D FE model using conventional modeling techniques. Accurate and efficient modeling can help to understand the complicated nature of a tooth that is surrounded by the jawbone. The success of modeling depends on the accuracy in simulating the geometry and surface structure of the tooth, the material characteristics of the tooth and jawbone, the loading and support conditions as well as the biomechanical tooth-jawbone interface. 

CT allows both the acquisition of bone morphology and measurement of bone density in a living individual. Thus, it is assumed that the data can be utilized for 3D FE modeling with material properties set precisely to reflect the detailed morphology and internal bone structures. Accordingly, 3D FE modeling from CT images is of great importance in understanding the individual simulations of stress distribution and stress values. In particular, issues have been addressed regarding enamel lesions, tooth loss, temporomandibular joint disorders, structural design of prosthetic appliances, and optimum implant planning. The elucidation of mechanical behavior utilizing industry practices can contribute to their resolution as well as medical reliability and safety.

 In this study, the 3D FE model of the lower first premolar, with the three layers of enamel, dentin, and pulp, and the mandible, with the two layers of cortical and cancellous bones, was directly constructed from noninvasively acquired CT images. This model was used to develop a system to analyze the stresses on the teeth and supporting bone structure during occlusion based on the FEM and to examine the possibility of mechanical simulation.

## 2. Materials and Methods

In the present study, a healthy young student in his twenties without caries who gave informed consent for participation and use of his mandible was included.

### 2.1. CT Scanning

CT scanners (Alphard Series Alphard-3030, the Asahi Roentgen Ind. Co., Ltd., Kyoto, Japan) were used to obtain the data necessary for the study. The imaging conditions are shown in Table 1. A total of 460 images were obtained, and the image data were saved as files in DICOM (medical imaging standard) format.

### 2.2. Reconstruction of 3D FE Models from CT Images

**Figure 1:**
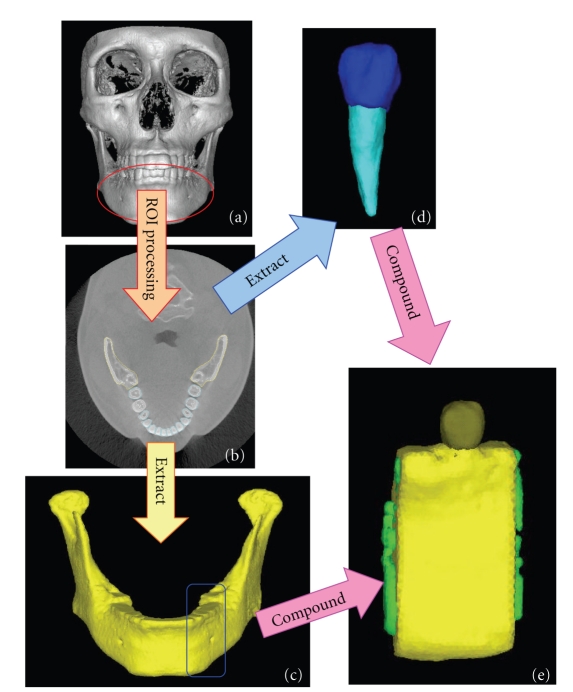
Flow of procedure for the FE modeling of the mandibular bone and tooth by MF. (a) CT scan of the testee, (b) extracting bone lines, (c) mandibular bone, (d) tooth, (e) 3D FE models.

Integrated software for digital image processing and finite analysis (Mechanical Finder MF ver. 5.1, Research Center for Computational Mechanics, Osaka, Japan) was used for reconstruction of the data converted to the DICOM specification file in the 3D FE model on the computer. The procedures for image processing and preparation of the analytical model are shown in Figure 1. Among the 460 images taken in 17 seconds, 239 CT images corresponding to the mandible used in this analysis were selectively input into the MF (Figure 1(a)). The criteria used ensured allowable exposure dose and a high resolution of 3D images of the mandible with teeth [17]. The region of the model to be reconstructed was extracted (region of interest (ROI) processing) (Figure 1(b)). The ROI processing consisted mainly of selection of a region of interest on each of the slices with or without hand work, and construction of a 3D model by integrating the region of interests marked on the slices. Processing the threshold value for gradation of the CT images enables automatic distinction of tissue and bone. As shown in Figure 1(b), the monitoring region was extracted by surrounding the target region. A cross-sectional photograph obtained by ROI processing was synthesized on the computer, and the 3D FE model was automatically reconstructed by superimposing images. However, corrections were made manually for areas where threshold value processing was insufficient because of similar threshold values. The 3D FE model used in this study was reconstructed as a 3D model by setting the threshold values required for classification on the 2D images because of the detailed classification of the cortical bone, cancellous bone, enamel, dentin and dental pulp, and by superimposing images from the boundary regions. A 3D FE model of the mandible with the mandible divided into two layers: the cortical bone and the cancellous bone (Figure 1(c)) and a 3D FE model of the mandibular premolars with the tooth divided into three layers: the enamel, dentin, and dental pulp layers were prepared (Figure 1(d)). One 3D FE model was completed by integrating these three models (Figure 1(e)).

### 2.3. Mesh Generation

The 3D FE model constructed from CT images was meshed. Outer mesh sizes for cortical bone and cancellous bone were set to 0.6 mm and 0.8 mm, respectively, and those of the enamel, dentin, and pulp were all set to 0.01 mm. The outer mesh was generated and checked, and subsequently the inner mesh was generated. The inner mesh size was set to range from a maximum of 5.0 mm to a minimum of 0.8 mm. The finer meshing was so beneficial in improving model accuracy that the mesh was generated in as small size as possible. However, according to the reduced mesh size, larger CPU capacity was required to correspond to the increased load on the CPU.

### 2.4. Material Property and Boundary Conditions

**Figure 2:**
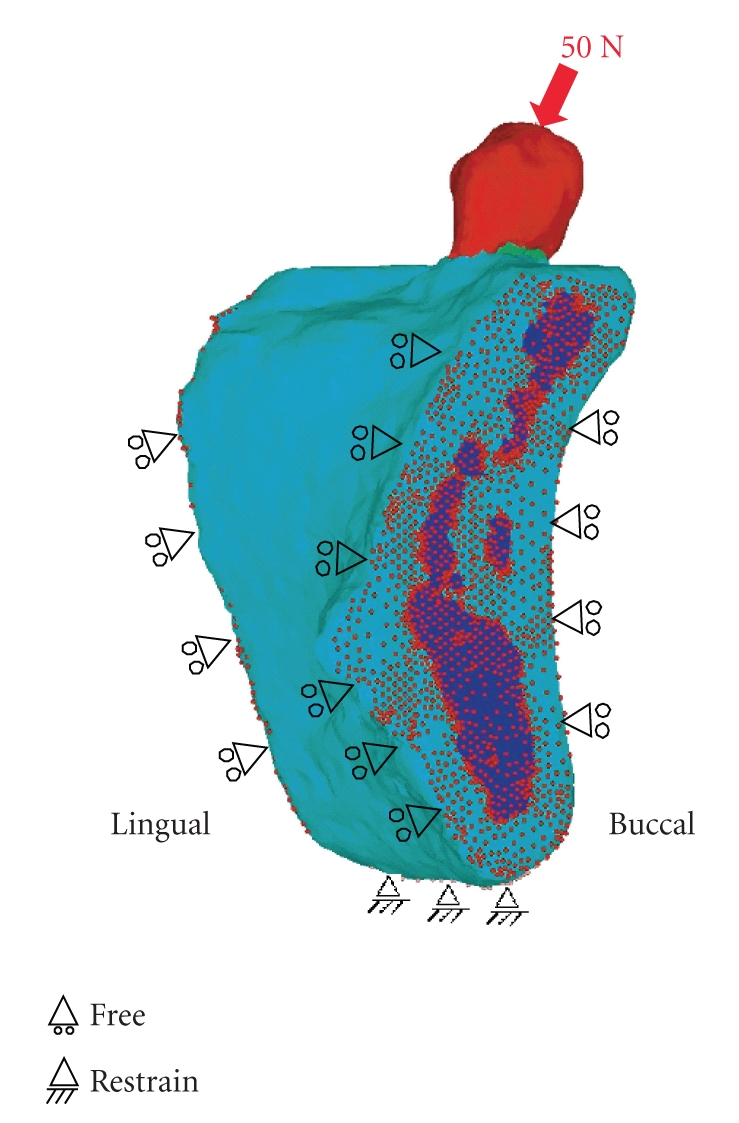
Geometrical and boundary conditions. A buccolingual force at a 45-degree oblique angle to the tooth axis was concentrated onto a single contact point, and static occlusal load of 50 N was applied to the premolar buccal cusp of teeth. Inferior border of the mandible was assumed to be fixed.

A static occlusal force F = (Fx, Fy, Fz) = (0, −35.4 N, −35.4 N) was applied to the edge of the upper tooth surface as shown in Figure 2. A buccolingual force at a 45° oblique angle to the tooth axis was concentrated onto a single contact point, and the intensity of the force was set to 50 N [18–21] to simulate the mean occlusal force during mastication on the implant-supported prosthesis. For the constrained boundary conditions, the whole inferior surface of the mandible was restrained in all degrees of freedom, and all medial and distal surfaces of the mandible were restrained only in X-axis translation and were free along the Y- and Z-axes. The analysis model consisted of cortical and cancellous bone, enamel, dentin, and pulp. The total numbers of nodes and elements (four-node tetrahedral element) comprising the model were 54,728 and 302,335, respectively. The elasticity modulus and Poisson's ratio [21–24] used for each part in this study were set as listed in Table 2.

### 2.5. Analysis Methods

Linear static analysis was implemented, and the material property of bone was assumed to be linearly elastic. A PC workstation (Precision Work Station 670, Dell Inc. Round Rock, TX, USA) was used for model construction and FE analysis. As for the results, maximum principal stress, a typical and commonly used failure criterion, was used for detailed evaluation of the model and determination of stress distribution in the cervical region of the teeth and adjacent jawbone.

## 3. Results

### 3.1. 3D FE Model

**Figure 3:**
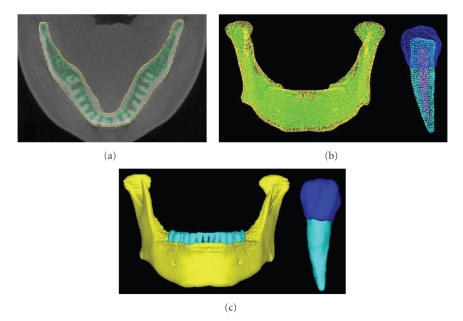
Geometry of 3D images and 3D models. (a) ROI of CT image, (b) 3D solid model, (c) 3D FE models.

The ROI extracted image is shown in Figure 3(a). The 3D solid model before element splitting and the 3D FE model are shown in Figures 3(b) and 3(c), respectively. The 3D solid model in Figure 3(b) reproduces the correct ROI extraction range and visual consistency with CT images was verified. The reproducibility of the extracted ROI regions and constructed 3D models was indirectly evaluated by quantitative comparison of the dimensions of standard block models constructed by the ROI procedure and those of the real blocks. The reproducibility of the blocks was good. The error did not exceed 1% [25]. The 3D model of the mandible was also examined for visual consistency. 

### 3.2. Stress Distribution of the 3D FE Model

**Figure 4:**
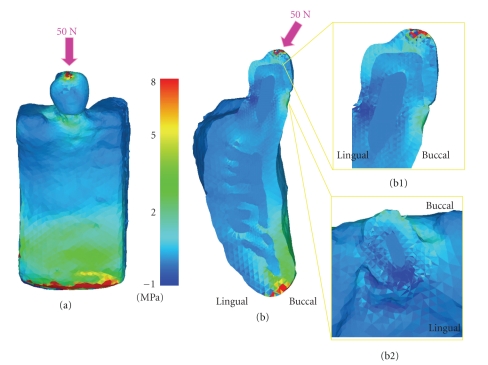
Maximum principal stress distribution. (a) Buccal view, (b) Buccolingual cross-section, (b1) Magnify of Buccolingual cross-section, (b2) Magnify of horizontal cross-section at the cervical.

The buccal view and buccolingual cross-sectional view of maximum principal stress are shown in Figures 4(a) and 4(b), respectively. In the buccal view, stresses of about 1.0–3.0 Mpa and about 5.0–7.0 Mpa were observed in the bone around the buccal cervical region of the teeth and the inferior border region of the mandible, respectively. In the buccolingual cross-sectional view, a stress of about 2.0–3.0 Mpa was present at the cortical bone around the buccal cervical region of the teeth and the buccal cervical enamel surface. In the buccolingual horizontal-sectional view around the cervical region, a stress of about 2.0–3.0 Mpa was observed on the buccal side of the enamel. Additionally, in the occlusal view in the coronal-apical direction, a stress of about 3.0 Mpa was detected around the enamel-dentin junction. The distributions of these stresses did not extend beyond the enamel-dentin junction but remained within the enamel (Figures 4(b1) and 4(b2)). In the region around the inferior border of the mandible, the stress was distributed to the cortical bone, while no significant stress distribution was observed for dentin, pulp, and cancellous bone. A high stress level of about 8.0 Mpa was demonstrated in the inferior region of the mandible whereas the tooth and bone were not under heavy stress.

## 4. Discussion

### 4.1. Regarding the Present Study

Most of the analysis in the previous mechanical studies was based on geometric morphology, and mean data were used for the modeling, which compromised precision. In mechanical analysis, precision of the model will presumably affect the results of the analysis. Therefore, precise construction of the model is required. Usually, modeling and analysis are performed with different software. Software that creates 3D mandible models, for instance, usually does not use bulk CT data. Some use micro-CT data, some use geometrical data taken from 3D shape measuring devices, and some simply use anatomic average dimensions. Analytical software, on the other hand, imports these geometrical data and creates FE models to analyze. Such procedures are really time consuming. 3D geometrical data so constructed sometimes cannot be imported into analytical software.

 The present software called Mechanical Finder (MF) has integrated software for modeling and analyzing. It can import bulk CT data directly from CT machines in the form of DICOM data and create 3D models to analyze. The procedures involved are very simple and not so time consuming. Since the software enables modeling and analyzing, the reproducibility of FE models is good with adequate consistency. The results of the analyses made on MF agree well with those obtained by another analytical software program called ANSYS [26].

 In addition, analysis with a model more approximate to living subjects may be a crucial factor in determining the direction of optimum treatment strategy with the help of mechanical simulation. The possibility of mechanical simulation to determine an optimum treatment plan for living individuals is indicated, and in light of safety and noninvasiveness, it seems to be very useful to investigate the mechanical influence using the 3D FEM. A single program for both analysis model construction and finite element analysis resulted in a more credible analysis than ever before.

### 4.2. Experimental Method

To perform the finite element analysis, the 3D FE model should be constructed within the computer, which is a time- and labor-inefficient process using traditional methods. In addition, the difficulty in constructing the 3D FE model for each living individual has obliged us to use a common 3D FE model constructed from anatomical mean values and data derived from dry skulls. In implant practice today, the development of a diagnostic system with the aid of mechanical analysis is required in order to attain high treatment predictability. To this end, a 3D FE model must be constructed that precisely reflects the variability of living subjects in a short time. CT imagery has been used in many implant treatments to provide essential diagnostic information. Therefore, in this study, a 3D FE model was constructed directly from CT images to reflect living individual variability. With the completed 3D FE model of the lower premolar combined with it, stress was analyzed. Accordingly, a stress analysis with the 3D FE model constructed to reflect living individual variability is now available, which indicates the high-level achievement of the two conflicting goals of analytical precision necessary for clinical application and the reduction of time. FE model construction is not an easy task; however, FEM is highly universal in the assessment of results and can feasibly simulate a model with a complex geometrical shape and allows precise measurement of internal stress. Therefore, the mechanical evaluation in this study used FEM to analyze stress. The values for principal stress or Von Mises stress are commonly calculated to evaluate stress in the FEM. The principal stress is frequently used as the failure criterion of brittle materials and distinguished by the direction and sign of the stress, which allow identification of either compressive stress or tensile stress. Von Mises stress is accepted as a useful failure criterion for ductile materials as well as many other kinds of materials.

 No function to construct the 3D FE model is included in the finite element analysis program commonly used, and CAD programs or special independent programs for 3D FE modeling have mainly been used for the modeling. However, with the program from this study, which allows 3D FE modeling from CT images of living subjects and measurement of the nonlinear material property of bone, which can be determined by employing different formula calculations, analysis with the 3D FE model reflecting the property of bone seems feasible. In the present paper, we describe general characteristics of a commercial program called MF. The program can perform nonlinear stress analyses of composite structures comprising of tissue and bone having different elastic stiffness values. These values vary with the density of the materials. In an ongoing investigation, the authors are analyzing the complex behavior of the composite mandible using the present FE code and the results will be reported elsewhere. As MF was used in this study and the operation proceeded in series, a binary threshold level was created during the ROI procedure. This operation allowed automatic extraction of the necessary regions according to the CT values. For bone, the fully reproducible target region could be extracted. However, automatic modeling with subdivisions of cortical bone, cancellous bone, and teeth (enamel, dentin, and pulp) is difficult due to their similar CT values. At present, manual correction is required for precise extraction of ROIs. In such cases, a longer time was needed compared to cases where only bone was the object. Therefore, further investigations for optimization of scanning conditions and refinement of CT images are needed to achieve automatic identification of living structures.

 The mesh size significantly affects the accuracy of the analysis. Not only the precision of the object model and material properties, but also the kinds, shape, and number of elements affect the accuracy of the analysis. Thus, careful consideration should be paid in element selection. There are many cases where the element size possibly deforms the morphology of the 3D FE model generated after division into elements. In this study, the element size was set to 0.20–5.00 mm on a side, and the element number was increased to 302,335 to allow reproduction of smooth 3D curves, which successfully and precisely reflected the curvilinear bone contexture. The element number can be theoretically increased to infinity depending on the processing capacity of the computer. A workstation with the maximum features currently available (CPU: Intel Xeon 3.6 GHz×2, memory capacity: 4 GB, HDD: 500 GB 3.0 Gb/s NCQ (7200 cycles) ×2) was used in this study. Any computer with higher performance may improve the precision of the 3D FE model and shorten the time required for construction.

 With the influence of external forces considered, the load was directed at a 45° buccolingual oblique angle downward to the tooth axis to simulate the direction of the load and force during lateral movement. The horizontally loaded occlusal force is supposed to be more destructive than a vertical force. The vertically loaded force is evenly distributed to the tooth and surrounding bone to cause little harmful effect. Conversely, the horizontally loaded force torques the tooth, exerting a stretching effect on bone, and thus has a harmful effect. In light of the horizontal loading during mastication speculated to be the greatest [27], such horizontal loading of the force was examined.

### 4.3. Analytical Results

The results of the stress analysis using the lower premolar 3D FE model revealed stress concentrated around the buccal cervical region, where the horizontal force was applied from the buccal side. The stress distribution observed in the cross-sectional view did not extend beyond the enamel-dentin junction but remained within the enamel. This concentration of stress around the buccal cervical region due to the horizontally loaded force may potentially cause wedge-shaped defects. Clinically, wedge-shape defects [28] are frequently encountered, but the cause has not yet been clarified. Black [29] explained that tooth brushing is one factor that causes the wedge-shaped defect. Miller [30] also reported that the main cause of the wedge-shape defect was the mechanical factor of tooth brushing, which has been supported by many investigators. However, recent studies suggest that repeated stress due to occlusal force may induce formation of these wedge-shaped defects. Lee et al. [31] compared the changes in the stresses with different occlusal loading sites and directions. This study showed the presence of tensile stresses in the cervical region of a maxillary premolar by various loading sites and in different directions. The results coincided with the stress-induced theory, hence sustaining it. The relationship of the affected factors of leverage to the development of cervical abfraction lesions was explored. The findings of the above study were confirmed in the present study.

## 5. Conclusions

In this study, the 3D FE model of the lower first premolar, with the three layers of enamel, dentin, and pulp, and the mandible, with two layers of cortical and cancellous bones, was directly constructed from noninvasively obtained CT images. This model was used to develop a system to analyze the stresses on the teeth and supporting jawbone during mastication based on FEM. The study was conducted to examine the possibility of mechanical simulation.

1. In this study, it was possible to prepare highly accurate 3D FE models from individual body CT images. A highly reliable simulation was possible by evaluation from a mechanical standpoint using the same model.
2. The results of the stress analysis using the 3D FE model of the lower premolar constructed with individual living X-ray CT images revealed stress concentration in the buccal cervical enamel and adjacent bone, and consistent reproduction of the stress distribution was achieved using the developed system.

With these results, the 3D FE model constructed with X-ray CT images can be used successfully for different mechanical simulations. Based on the present study, a novel mechanical clinical diagnostic criterion could be clinically applied in individual cases for virtual treatment simulation and stress analysis.

## Figures and Tables

**Table 1:** Present exposure conditions.

Tube voltage	80 kV
Tube current	5 mA
Voxel size	0.39 mm
Exposure time	17 s
Exposure mode	C-mode

**Table 2:** Material properties of mandibular bone (compact bone, spongy bone) and teeth (enamel, dentin, pulp).

	Young's modulus (MPa)	Poisson's ratio
Enamel	4.80 × 10^4^	0.23
Dentin	1.80 × 10^4^	0.31
Pulp	2 × 10	0.45
Compact bone	1.30 × 10^4^	0.30
Spongy bone	0.13 × 10^4^	0.30
